# Multifunctional Platforms Based on Graphene Oxide and Natural Products

**DOI:** 10.3390/medicina55060230

**Published:** 2019-05-30

**Authors:** Alexa Croitoru, Ovidiu Oprea, Adrian Nicoara, Roxana Trusca, Mihai Radu, Ionela Neacsu, Denisa Ficai, Anton Ficai, Ecaterina Andronescu

**Affiliations:** 1 Academy of Romanian Scientists, Spl. Independenței 54, 50085 Bucharest, Romania; croitoru.alexa@yahoo.com (A.C.); adrian.nicoara@upb.ro (A.N.); radu_mihai2009@yahoo.com (M.R.); ionela.neacsu@upb.ro (I.N.); ecaterina.andronescu@upb.ro (E.A.); 2 University Politehnica of Bucharest, Faculty of Applied Chemistry and Materials Science, Gh. Polizu St 1-7, 011061 Bucharest, Romania; ovidiu.oprea@upb.ro (O.O.); truscaroxana@yahoo.com (R.T.); denisa.ficai@upb.ro (D.F.)

**Keywords:** graphene oxide, nanomaterials, active substances, gallic acid, drug delivery

## Abstract

*Background and objectives:* In the last few years, graphene oxide has attracted much attention in biomedical applications due to its unique physico-chemical properties and can be used as a carrier for both hydrophilic and/or hydrophobic biomolecules. The purpose of this paper was to synthesize graphene oxide and to obtain multifunctional platforms based on graphene oxide as a nanocarrier loaded with few biologically active substances with anticancer, antimicrobial or anti-inflammatory properties such as gallic acid, caffeic acid, limonene and nutmeg and cembra pine essential oils. *Materials and Methods:* Graphene oxide was obtained according to the method developed by Hummers and further loaded with biologically active agents. The obtained platforms were characterized using FTIR, HPLC, TGA, SEM, TEM and Raman spectroscopy. *Results:* Gallic acid released 80% within 10 days but all the other biologically active agents did not release because their affinity for the graphene oxide support was higher than that of the phosphate buffer solution. SEM characterization showed the formation of nanosheets and a slight increase in the degree of agglomeration of the particles. The ratio I_2D_/I_G_ for all samples was between 0.18 for GO-cembra pine and 0.27 for GO-limonene, indicating that the GO materials were in the form of multilayers. The individual GO sheets were found to have less than 20 µm, the thickness of GO was estimated to be ~4 nm and an interlayer spacing of about 2.12 Å. Raman spectroscopy indicated that the bioactive substances were adsorbed on the surface and no degradation occurred during loading. *Conclusions:* These findings encourage this research to further explore, both in vitro and in vivo, the biological activities of bioactive agents for their use in medicine.

## 1. Introduction

Carbon is an extraordinary element which can exist in different allotropic forms. It is found in large quantities in nature as coal or as natural graphite and in fewer quantities as diamond. The crystal structure of diamond has a cubic arrangement of the atoms compared to graphite which has a special kind of structure containing flat sheets of carbon atoms bonded into hexagonal structures. The later, has remarkable properties, with good thermal and electrical conductivity [1] and starting from these hexagonal structures new engineered materials can be obtained such as graphene and carbon nanotubes.

**Figure 1:**
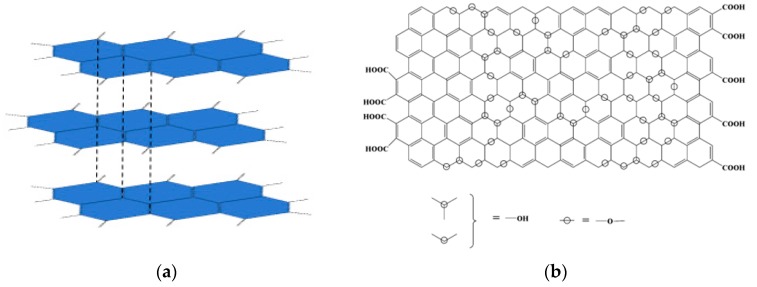
(**a**) Graphite structure highlighting containing graphene sheets and (**b**) Graphene oxide structure [32].

Carbon nanomaterials contain many different allotropic forms of carbon, the most studied and used ones being carbon nanotubes and graphene. The remarkable properties of graphite (Figure 1a) are even enhanced, or even new properties are obtained once the three-dimensional arrangement of the graphite turns into the bidimensional structure of graphene. After their discovery, they have become very important for researchers because of their specific physical, chemical, mechanical and electrical properties such as chemical and thermal stability and electrical conductivity, thus having applications in biomedical and biological fields [2,3,4,5]. 

Inorganic nanomaterials have made significant progress in the medical field, including cancer therapy, imaging, drug delivery and soft tissue repair and regeneration. Inorganic nanomaterials have been used for improving stem cell engraftment in cellular therapy, the mechanical stability of materials in tissue repair, electrical conductivity in nerve, cardiac regeneration and antibacterial activity in wound dressings. These nanomaterials have also been used to improve or replace common surgical materials and restore functionality to damaged tissue [6].

They have a great potential in biomedical applications including molecular imaging, cancer and gene therapy, drug delivery, biosensors and tissue engineering applications. In medical applications, carbon nanotubes have the ability to penetrate different body tissues and to carry large doses of agents, providing therapeutic effects. Contrast agents associated with carbon nanotubes improve cell and tissue visualization and so new pathological development patterns can be detected. However, various factors and parameters of carbon nanotubes (CNT) including the size, length, shape, surface, agglomeration, doping or functionalization can cause toxicity through oxidative stress [7] or contrarily, to induce improved performances [8,9].

Due to their nanometric structure and remarkable properties, carbon-based materials are studied for biomedical applications. Significant progress has been made to overcome some of the major obstacles in biomedical applications of nanomaterials, especially when it comes to water, namely dispensability and safety issues. A great disadvantage is their insolubility in many solvents, especially in water, carbon nanotubes, as well as graphene sheets, being incompatible with biological systems, but their hydrophilic/hydrophobic behavior can be easily controlled by oxidation. Their toxicity is also a major drawback due to water insolubility and potential metallic impurities (especially for CNT, the metallic impurities being related to the used catalysts). Thus, the functionalization of carbon-based materials is essential in order to become soluble and to have minimal toxicity for biomedical applications [10,11,12]. Usually, functionalization involves two or even more steps, firstly surface oxidation (with the generation of hydroxyl and carboxyl groups) followed by physical (adsorption) or chemical attachment (via covalent bonding) of more biocompatible moieties [13,14,15,16,17,18]. 

Graphene oxide (Figure 1b) is considered to be a promising material for biomedical uses due to its unique physical and chemical properties such as specific surface area, abundant oxygen-containing functional groups, good biocompatibility, electrostatic and hydrophobic interactions with drugs, mechanical stiffness, thermal stability, electrical and optical properties. Although several problems remain to be solved, the biological applications of graphene derivatives have important potential due to many attempts with promising results in the biofunctionalization and standardization of graphene derivatives, by fractionation based on size, the number of layers and chemical functionalities, but also by investigating long-term toxic effects [19,20]. Morales-Narvaez et al. [21] introduced a progress report regarding the behavior of GO as an optical biosensing system. GO plays an important role in producing cost-effective devices, for example by simplifying manufacturing processes and saving expensive bioreagents due to its outstanding physicochemical properties. In 2013, Bianco. and Wick along with their colleagues recommended a specific nomenclature and classification for 2D carbon materials, mainly based on their lateral size, number of layers, and oxidation degree. The significance of this features of GO is still little explored and in addition, heterostructures based on graphene derivatives and other 2D materials are expected to bring breakthroughs in biosensing as they enhance the light–matter interaction [22,23].

Graphene oxide and its derivatives have applications in various fields summarized in four main categories, controlled release and cancer treatment, biosensors, bio-imaging, and antibacterial activity [24,25,26]. The amphiphilic and planar structure of the graphene oxide (containing different functional groups: epoxy, hydroxyl, carboxyl, carbonyl, etc.) allows it to incorporate hydrophilic and/or hydrophobic biomolecules onto the structure in order to improve its stability in a particular environment and to have minimal toxicity for biomedical applications [13,27,28,29,30,31].

As the side effects of synthetic substances on the body are well known, lately there has been a great interest in using natural substances and in recent years great emphasis has been placed on the development of nanomaterials loaded with natural extracts [33]. There is an urgent need for controlled target delivery for cancer therapy, and synthesis under ecological conditions is important for the biomedical use of controlled release systems in the human body [34]. The antimicrobial, anticancer and antifungal effects of natural compounds extracted from different plants against various microorganisms have been demonstrated over time by researchers, which encourages us to further explore the biological activities of bioactive substances for their use in medicine [35,36,37,38,39]. 

**Figure 2:**
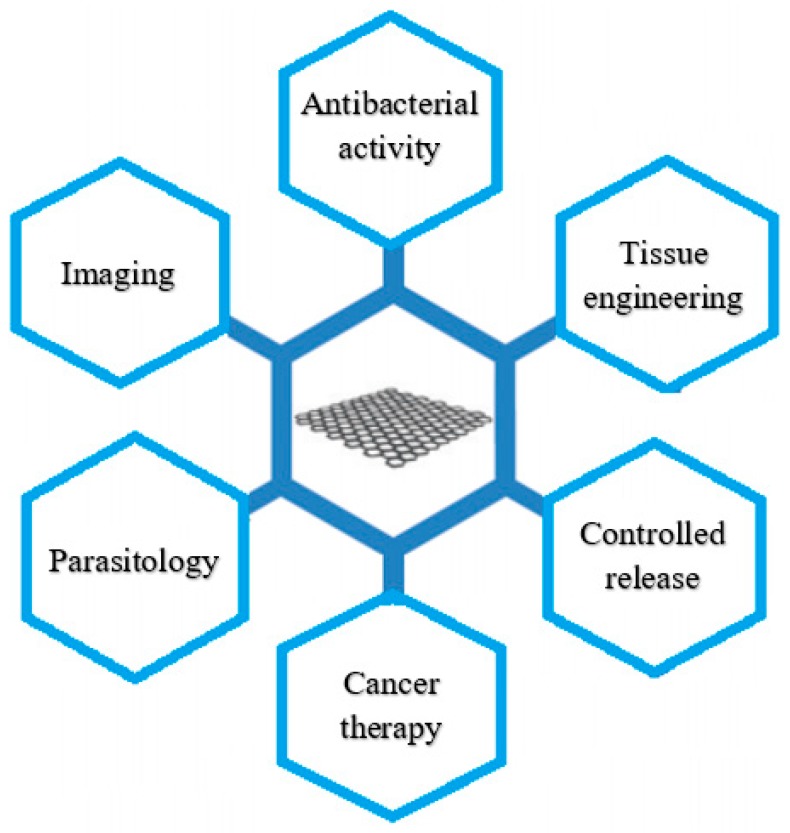
Graphene oxide applications [19].

Graphene oxide has an easily modified surface with a variety of biocompatible polymers such as chitosan, polyethylene glycol, poly-L-lysine and polyvinyl alcohol. Graphene oxide contains a large amount of marginal hydrophilic groups. These moieties make graphene oxide more attractive for researchers with applications in controlled release, parasitology, tissue engineering, antibacterial activity, cancer treatment, imaging, sensors and diagnostics. The most important applications of graphene oxide are shown in Figure 2. In order to use graphene oxide in clinical trials, it is essential to study its toxicity and biocompatibility through in vitro and in vivo experiments using specific cell lines and animal models [27,40].

Despite advances in technology in biomedical science, cancer still remains one of the greatest challenges for humanity. In this paper, we wanted to obtain a nanostructured platform for controlled release system using graphene oxide as a nanocarrier for several active agents with anticancer properties as well as antimicrobial and anti-inflammatory properties. The active compounds used were gallic acid, caffeic acid, limonene and two essential oils: nutmeg and cembra pine oil.

## 2. Materials and Methods

Graphite powder (99.99%), potassium permanganate (99.22%) (Lach-ner, Neratovice, Czechia), sulphuric acid (95–97%) (Merck, Darmstadt, Germany), hydrochloric acid (36.5–38%) (Silal Trading, Bucharest, Romania), phosphorus pentoxide (≥98%) (Sigma-Aldrich, Steinheim, Germany), potassium peroxodisulfate (≥98%) (Sigma-Aldrich), hydrogen peroxide (35%) (Silal Trading), gallic acid (≥98%) (Merck), caffeic acid (≥98%) (Sigma-Aldrich), limonene (≥95%), cembra pine and nutmeg essential oils by Carl Roth, Karlsruhe, Germany and ethyl alcohol (96%) (Chimreactiv, Bucharest, Romania) were used in this paper. 

### 2.1. Synthesis of Graphene Oxide

**Scheme 1:**
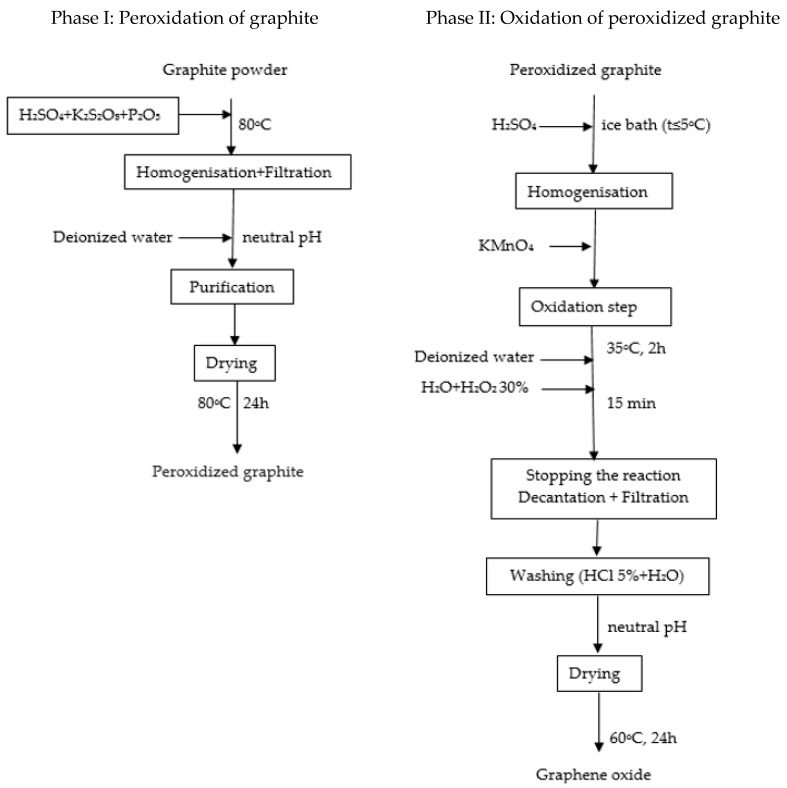
Synthesis of graphene oxide.

Graphene oxide was synthesized from graphite powder using a modified Hummers method presented in Scheme 1 [41]. In this method, a solution containing concentrated H_2_SO_4_ (60 mL), K_2_S_2_O_8_ (10 g) and P_2_O_5_ (10 g) was heated at 80 °C. Then, 20 g of graphite was added under stirring condition. The solution was left to cool down to room temperature. Then, a large amount of deionized water was added, and the solution was filtered, and the filtrate was rewashed until reached neutral pH. The washed product was dried for 24 h at 80 °C. Concentrated H_2_SO_4_ (460 mL) solution and 20 g of peroxidized graphite were added under stirring condition in a Berzelius flask in an ice bath (temperature below 5 °C). Sixty gram of KMnO_4_ was then added slowly into the solution and stirred until the solution became dark green. The solution was then transferred to a beaker, where it was stirred for 2 h at 35 °C. About 920 mL of distilled water was added to the mixture and stirred for 15 min, after which 2.8 L of distilled water and 50 mL of 30% H_2_O_2_ were added to eliminate the excess of KMnO_4_. The supernatant was decanted, and the residuals were then rewashed with 5% HCl and distilled H_2_O until a neutral pH was obtained. The washed GO suspension was dried at 60 °C for 24 h.

### 2.2. Loading of Biological Active Agent (BAA)

In order to obtain graphene oxide—gallic acid (GOGA), a 1% solution of gallic acid was prepared. From this solution, three other solutions were made in a 10 mL volumetric flask, adding 0.1 g graphene oxide in each solution. The mixtures were stirred for 16 h and the GOGA nanomaterial was collected via filtration and dried in a vacuum oven at 40 °C for 24 h. 

The same method was used to load graphene oxide with caffeic acid, limonene, cembra pine and nutmeg essential oils (according to Table 1). The controlled release of the biologically active agents loaded onto the GO platforms was studied using a buffer solution of pH 7.4 (similar to the pH of blood).

To study the controlled release of the loaded biological active agent (BAA) onto graphene oxide, a phosphate buffer solution with a pH of 7.4 was used. Various studies showed that anions such as ${\mathrm{HPO}}_{4}^{2-}$ and $H_{2}{\mathrm{PO}}_{4}^{-}$ influence the controlled release of drugs [54,55]. The release rate of the BAA was measured by adding 65 mg GO-BAA into 25 mL of phosphate buffer solution (pH = 7.4). From time to time (1, 4, 24, 48, 123 h, respectively, and 10, 21 and 30 days) aliquots were removed and analyzed in order to evaluate the released amount of the biologically active agents. During this time, the solutions were kept in the oven at 37 ± 1 °C. After removal, the solutions were centrifuged for 10 min at 8000 rpm and the clear solutions were analyzed by HPLC.

HPLC analysis was conducted on Agilent 1260 Infinity liquid chromatograph (Agilent, Santa Clara, CA, USA) equipped with a DAD detector. The analytical column used was Eclipse Pluse C_18_ (4.6 mm × 100 mm, 3.5 µm). Two-gradient elution system was used: mobile phase A contained water and 0.1% formic acid and mobile phase B was acetonitrile with 0.1% formic acid. The mobile phase composition was 50:50. Elution was performed at a solvent flow rate of 1 mL/min. The sample injection volume was 15 µL and peak detection was at 220 nm. The retention time was 0.85 min. 

The synthesized products were characterized by FTIR using a Nicolet iS50FT-IR (Nicolet, MA, USA) spectrometer equipped with a DTGS detector which provides information with a high sensitivity in the range of 4000 cm^−1^ and 100 cm^−1^ at a resolution of 4 cm^−1^.

Raman spectroscopy analyses were performed using a Horiba equipment (Labram HR Evolution, Pailaiseau, France) with an excitation wavelength of 514 nm and a 50X objective with a 10 s acquisition time. 

Thermogravimetric analysis was performed using an STA TG/DSC Netzsch Jupiter 449 °C equipment (Selb, Germany), with the temperature ranging between 25 and 900 °C in a dynamic atmosphere of 50 mL/min air with a heating rate of 10 K/min in an alumina crucible (Al_2_O_3_).

Scanning electron microscopy images were obtained using a high-resolution electron microscope equipped with a field emission electron source (FEI Inspect F50, Eindhoven, Nederlands) with a resolution of 1.2 nm at 30 kV and 3 nm at 1 kV (BSE). 

The transmission electron micrographs were obtained by using a Tecnai G^2^ F30 S-TWIN high-resolution transmission electron microscope (HRTEM, ThermoFisher, Eindhoven, Nederlands) equipped with STEM with HAADF detector, EDX, PEELS, energy filter, GIF operated at 300 kV. The sample preparation was done as follows: a small amount of GO was placed into water in a centrifuge tube and sonicated for 15 min. After that, 10 microliters of dispersed sample were placed onto 400 mesh holey carbon-coated Cu grid and left to dry prior to the transmission electron microscopy (TEM) analysis.

Zeta Potential was obtained using dynamic light scattering technique (DLS, DelsaMax Pro, Backman Coulter, Brea, CA, USA). The nanoparticle suspensions in ultrapure water were homogenized at room temperature using an ultrasonication probe for a period of 10 min.

### 2.3. Analysis of Functional Groups of Graphene Oxide

The Boehm method was used to analyze the content of functional groups in GO. Graphene oxide (0.1 g) was immersed into 100 mL of NaOH, NaHCO_3_ and 0.1M Na_2_CO_3_ separately. The base mixtures containing GO were magnetically stirred for 24 h and then filtered to separate GO sheets from solution. From the obtained extracts, 5 mL was titrated with 0.05 M HCl solution using phenolphthalein as an indicator. For proper interpretation of the results, blank samples were also performed without GO, having the same concentrations [56,57]. 

The concentration of various types of functional groups was calculated assuming that NaHCO_3_ neutralized only the carboxylic groups, Na_2_CO_3_ neutralizes the carboxylic groups and lactones and NaOH neutralizes all the carboxylic, phenolic and lactonic groups [58].

## 3. Results

This report presents the analyses of the five nanostructured platforms obtained by loading graphene oxide with biologically active agents: gallic acid, caffeic acid, limonene, nutmeg and cembra pine essential oils. 

The content of carboxyl group was 0.45 meq/g, of phenolic groups 0.57 meq/g and of lactones 0.15 meq/g, the total number of functional groups was 1.17 meq/g. The content of phenolic groups was higher as they play an important role in the structure of the graphene oxide [58].

Zeta potential, an indicator of the stability of the sample, is around −37.36 mV, which shows a good stability of the suspension [59].

**Figure 3:**
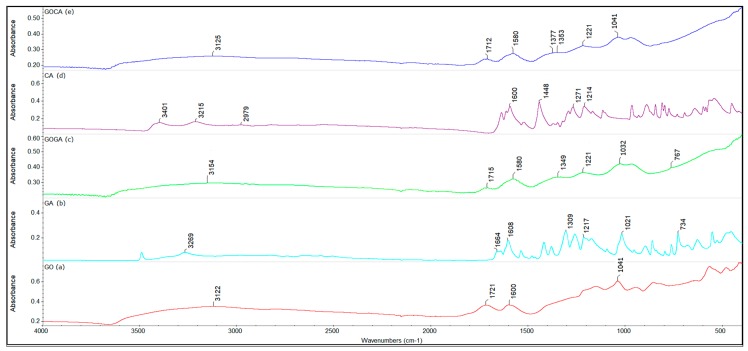
FTIR spectra for (**a**) graphene oxide (GO), (**b**) gallic acid (GA), (**c**) GOGA (gallic acid loaded onto GO nanocarrier), (**d**) caffeic acid (CA) and (**e**) GOCA (caffeic acid loaded onto GO nanocarrier).

Figure 3 presents Fourier Transform Infrared Spectroscopy (FTIR) spectra for graphene oxide, gallic acid, caffeic acid and gallic and caffeic acid loaded onto GO nanocarrier.

**Figure 4:**
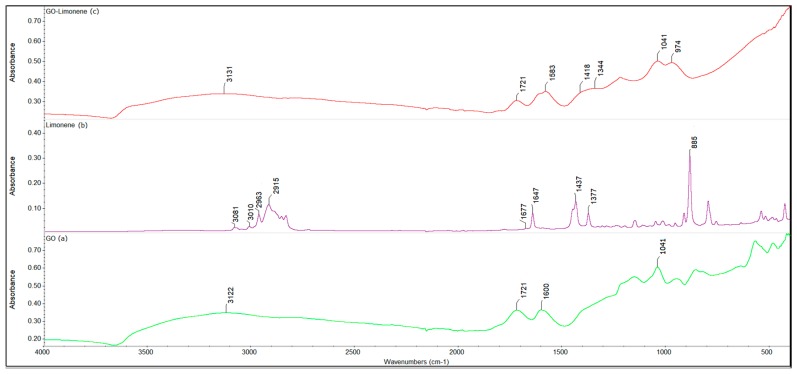
Fourier Transform Infrared Spectroscopy (FTIR) spectra for (**a**) graphene oxide (GO), (**b**) limonene and (**c**) graphene oxide loaded with limonene.

Figure 4 shows FTIR spectra for graphene oxide, limonene and graphene oxide loaded with limonene.

**Figure 5:**
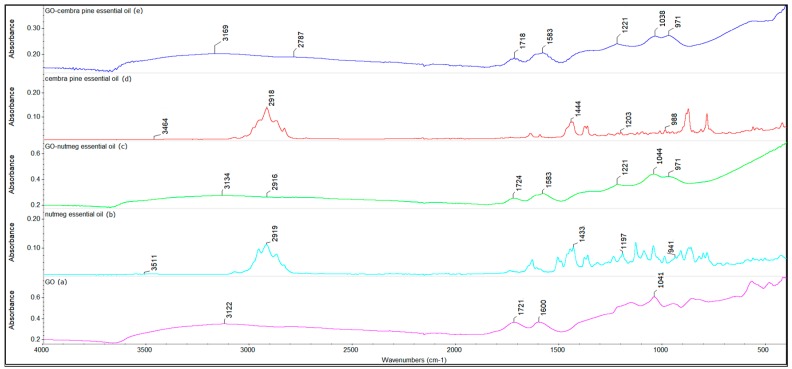
FTIR spectra for (**a**) graphene oxide (GO), (**b**) nutmeg essential oil, (**c**) graphene oxide loaded with nutmeg essential oil, (**d**) cembra pine essential oil and (**c**) graphene oxide loaded with cembra pine essential oil.

Figure 5 presents FTIR spectra for graphene oxide, nutmeg and cembra pine essential oils and graphene oxide loaded with nutmeg and cembra pine essential oils. 

**Figure 6:**
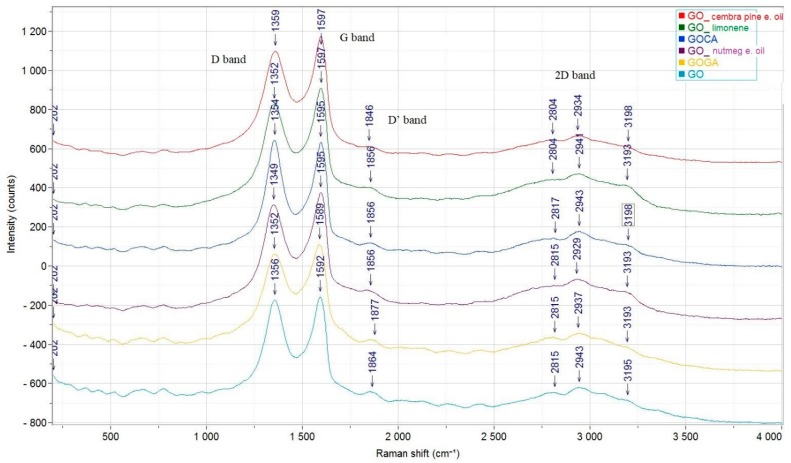
Raman spectra of GO_cembra pine oil, GO_limonene, GOCA, GO_nutmeg oil, GOGA and GO.

Raman spectroscopy was used to analyze the disorder and defects in the structure of graphene oxide and functionalized graphene oxide (Figure 6).

**Figure 7:**
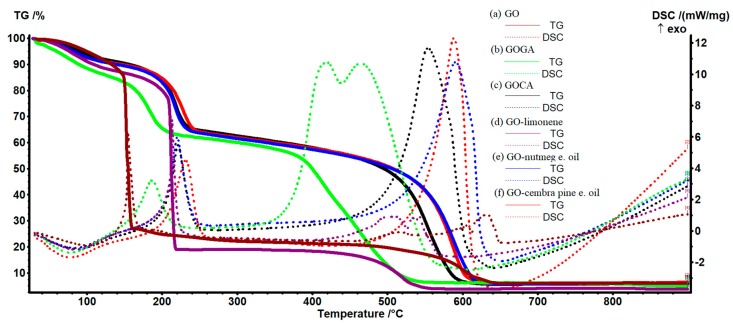
Thermogravimetric analysis of (**a**) graphene oxide, (**b**) GOGA, (**c**) GOCA, (**d**) GO-limonene, (**e**) GO-nutmeg essential oil and (**f**) GO-cembra pine essential oil.

Figure 7 presents the thermogravimetric analysis for graphene oxide, GOGA, GOCA, GO-limonene, GO-nutmeg essential oil and GO-cembra pine essential oil.

**Figure 8:**
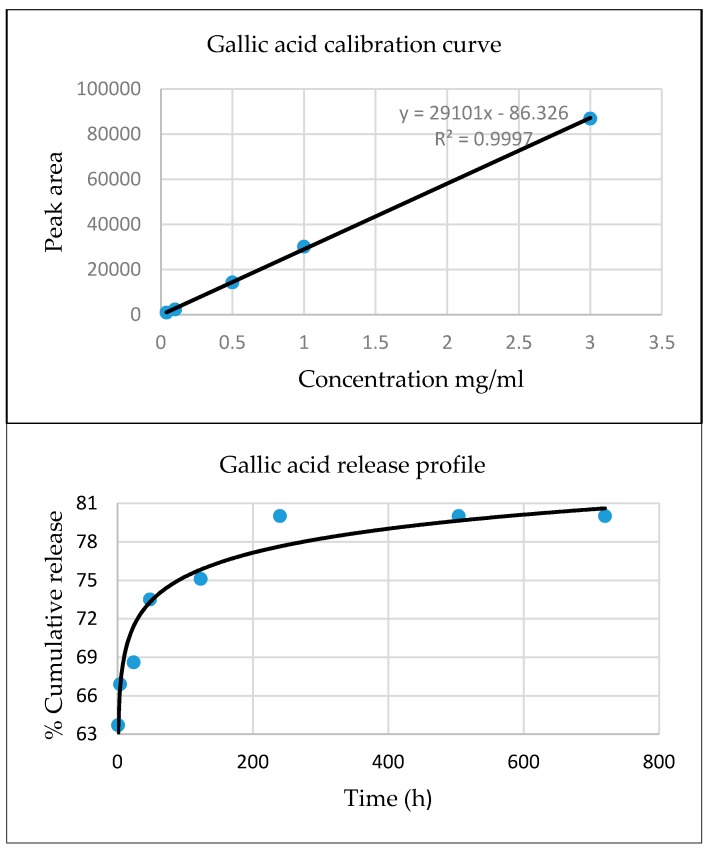
The calibration curve and the release profile for gallic acid.

The release behavior of gallic acid loaded onto graphene oxide is presented in Figure 8. 

**Figure 9:**
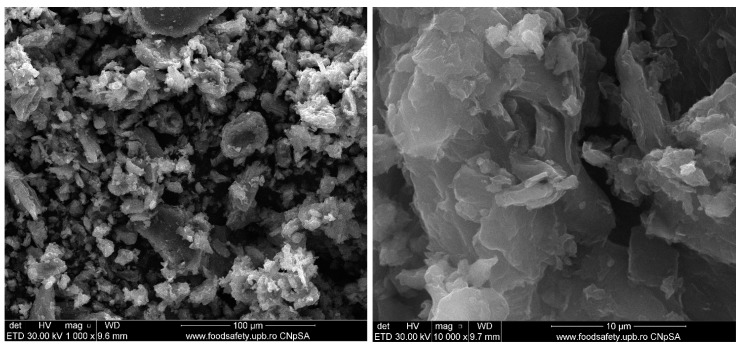

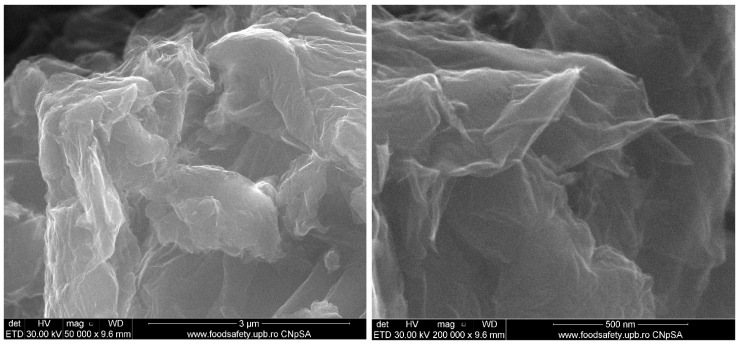
Scanning electron microscopy of the graphene oxide support.

**Figure 10:**
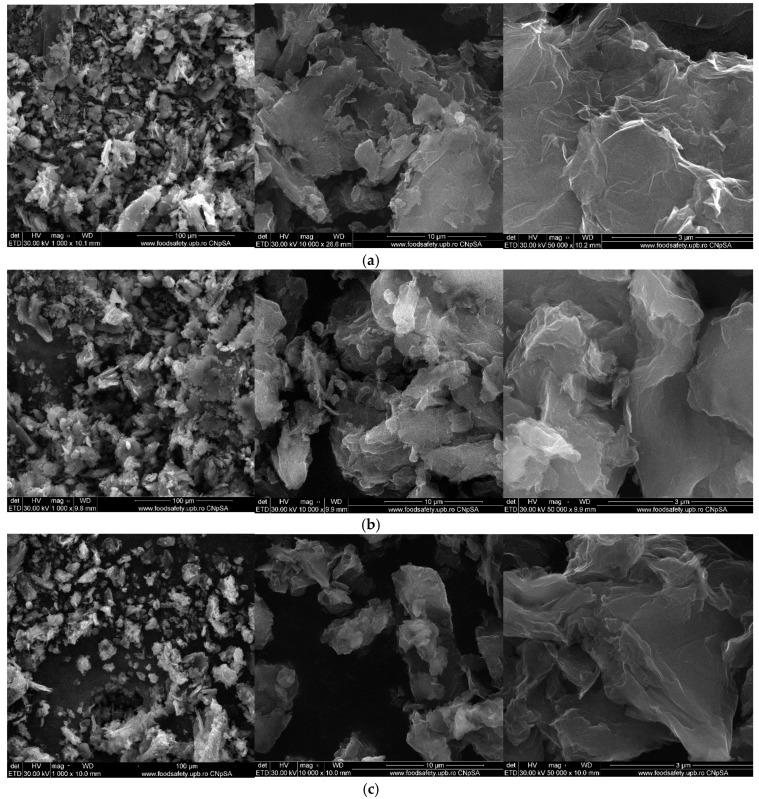

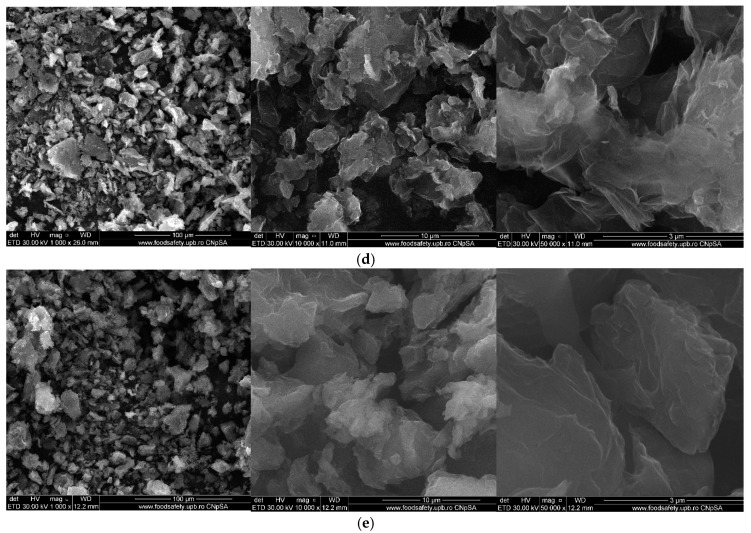
Scanning Electron Microscopy (SEM) image of the samples: (**a**) GOGA, (**b**) GOCA, (**c**) GO limonene, (**d**) GO nutmeg essential oil, (**e**) GO cembra pine essential oil.

Figure 9 and Figure 10 show the morphology of graphene oxide at 1000X, 10kX, 50kX and 200kX magnifications and of graphene oxide loaded with gallic acid, caffeic acid, limonene and nutmeg and cembra pine essential oils, respectively.

**Figure 11:**
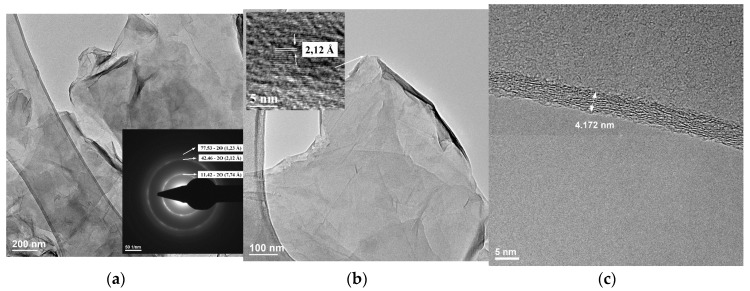
Transmission electron microscopy (TEM) image and selected area electron diffraction (SAED) pattern—insert (**a**), TEM image with a high-resolution transmission electron microscope (HRTEM) image—insert (**b**) and lateral view of the graphene layer indicating the thickness (**c**) of GO sheets.

Figure 11 shows representative TEM images at low and high magnification as well as the representative selected area electron diffraction (SAED) pattern (insert in image a), as well as a lateral view of the graphene oxide sheet.

## 4. Discussion

In several papers, these biological substances were studied for their benefits in the human body. Gallic acid has many biological applications such as antibacterial, antimutagenic, antiviral and antitumoral [43]. Based on the related research reports, caffeic acid has a variety of pharmacological effects, including anti-mutagenesis, anticancer, antibacterial, cardiovascular, anti-leukemic and immunomodulatory [46]. D-limonene is widely used in cosmetics, in the food industry, but also in pharmaceutical and medical applications. It has antioxidant, antimicrobial, anti-tumor, and antidiabetic properties [48,49]. Essential oils have traditionally been used for respiratory infections and are currently used as drugs for various diseases such as cardiovascular disease, diabetes, Alzheimer’s and cancer. Furthermore, studies have demonstrated the synergistic effect of ingredients in essential oils against various human pathogens [51,52]. 

### 4.1. Fourier Transform Infrared Spectroscopy (FTIR)

The resulting graphene oxide is water dispersible, the brownish aspect of the suspension is maintained even after 1 day, with only limited deposition after 1 day (~1g/L). As shown in Figure 3a, FTIR spectra of GO shows the characteristics peaks of graphene oxide. The peak from 1721 cm^−1^ is assigned to C=O stretching vibration present in the carbonyl and carboxyl groups of GO. The presence of the spectral band from 1600 cm^−1^ is attributed to C=C stretching vibration and the signals at 1224 cm^−1^ and 1029 cm^−1^ are corresponding to COC (epoxy) and COH (alcohol) stretching vibration groups. The broad peak at 3122 cm^−1^ is corresponding to the associated OH (hydroxyl) group from GO but also due to the adsorbed water [28,31].

Figure 3b shows characteristic bands of pure gallic acid, the signal at 1664 cm^−1^ indicates the presence of the phenolic group, and the signal at 1608 cm^−1^ is assigned to stretching vibration of C=C bonds of the aromatic ring. The stretching vibration of the carboxyl groups occurs at 1217 cm^−1^ and the signal at 1021 cm^−1^ is attributed to C-O stretching vibration of the carboxyl group. The peak at 734 cm^−1^ corresponds to the δ CC benzene ring vibrations. The signal at 3269 cm^−1^ corresponds to the OH stretching vibration.

After the loading of graphene oxide with gallic acid, the characteristic bands of the components can be highlighted. The infrared spectrum of graphene oxide loaded with gallic acid (GOGA) shows the characteristic signals of both graphene oxide and gallic acid, suggesting the successful loading. Certainly, some of these bands being overlapped, instead of individual peaks a composed peak can be observed, the intensity is proportional with the sum of the individual peaks. As the peaks from 1715 cm^−1^ and 1580 cm^−1^ are characteristic to GO, in Figure 3c these peaks can be visualized but the relative intensity is lower. The signal at around 3154 cm^−1^ corresponds to the OH group from carboxylic and phenolic groups. By subtracting between the GOGA nanocomposite spectrum and the pure graphene oxide spectrum a visible shifting and broadening of the band around 1850 cm^−1^ can be observed, but also an increase in the relative intensity of the peak, also highlighting the interaction of the acid gallic with the graphene oxide, which act as a promising nanocarrier for this BAA. The same can be observed for the peak from 1030 cm^−1^ [43,60].

In Figure 3d, the stretching vibration of the CH groups can be highlighted at a wavelength of 2979 cm^−1^. The strong intensity band from 1600 cm^−1^ is attributed to the stretching vibration of C=C bonds present in the carboxyl groups and the signal at 1448 cm^−1^ is attributed to C-C stretching vibration of the aromatic ring. The signals at 1214 cm^−1^ and 1071 cm^−1^ are corresponding to the stretching vibration of the C-OH bonds attached to the aromatic ring. The broad band between 3200 cm^−1^ and 3500 cm^−1^ corresponds to the vibration of the associated hydroxyl groups [61]. The two spectra of GO and GOCA are similar, the latter showing characteristics bands/signals of caffeic acid overlapped on the graphene oxide support. As shown in Figure 3e, differences are seen in the same areas of the spectrum, 1580 cm^−1^ and 1041 cm^−1^ indicating interactions between CA and GO. 

Figure 4b shows the characteristic bands of limonene. The bands from 1647 and 1677 cm^−1^ are assigned to the stretching vibration of C=C bonds of the aromatic ring in the vinyl group. The signals between 3010 cm^−1^ and 3081 cm^−1^ are assigned to the stretching vibration of the C-H bonds. The signals below 3000 cm^−1^ are generally associated with unsaturated groups. The two peaks at 1377 cm^−1^ and 1437 cm^−1^ are assigned to the bending CH_3_/CH_2_ bonds, while the intense signal at 885 cm^−1^ may correspond to the CH=CH bending. Figure 4c shows the characteristic peaks of limonene, but these are shifted and broadening (especially the band from 1583 cm^−1^) due to the interaction between the support and the limonene. Also, a difference in relative intensity between the two peaks from 1583 cm^−1^ and 1721 cm^−1^ can be observed because of the condition of the characteristics peaks of limonene and GO [62].

Based on the literature data [63] the main chemical compounds from nutmeg essential oil are sabinene, α-pinene and myristicin. Figure 5b shows similar signals to those identified by other researchers, such as the peaks at 941 cm^−1^ (corresponding to the CH stretching vibration) and at 1197 cm^−1^ (corresponding to the CO stretching vibration). The bending vibration of CH_3_/CH_2_ groups occurs at 1433 cm^−1^. In addition, the signal at 3511 cm^−1^ corresponds to the associated hydroxyl group, and the signal at 2919 cm^−1^ is attributed to the stretching vibration of the C-H bonds. In Figure 5c, the nanocomposite contains characteristic signals of essential nutmeg oil which demonstrate the loading of certain compounds on graphene oxide nanocarrier [64,65]. The FTIR spectra of essential oils are similar when it comes to the loading of active substances onto the graphene nanocarrier, resulting in the successful formation of the complex [66].

### 4.2. Raman Spectroscopy

The Raman spectrum (Figure 6) for the graphene oxide is similar to the spectra presented in previous works [67,68,69]. The intensity ratio corresponding to D band and G band can be used as a measure of the disorder in graphene oxide this band being accentuated by the functional groups of oxygen in the material [70]. An increase of ration of the band’s intensity indicates an increase of the disorder from the material. The D-band from graphene oxide is observed at 1356 cm^−1^, while the G band is noticed at 1592 cm^−1^ [70,71].

Comparing the spectra, structural changes occurred during the loading of GO with the active compounds. The G band, specific for the sp^2^ carbon forms, is observed at 1589 cm^−1^ for graphene oxide loaded with GA. The D band, specific for the sp^3^ carbon forms can be seen at ~1350 cm^−1^. The 2D band appears in the 2800 cm^−1^ and 3200 cm^−1^ range. Raman spectra did not show important differences in the D and G band between GO and GO loaded with bioactive compounds spectra, [43,72,73] thereby the graphene structure has not been altered, the bioactive compounds being adsorbed on the surface and no additional disorder was induced. The intensity ratio (I_D_/I_G_) for GO (0.97) is slightly larger than GO loaded with bioactive substances starting from 0.87 (GO-limonene) to 0.92 (GO-gallic acid) which suggests that the active compounds can repair the defects of graphene during functionalization [73].

In addition, from the Raman spectroscopy, the ratio between the 2D and G band is a method for determining the number of layers presented in the samples. The shape, position and intensity of the 2D band show the difference between single and multilayer graphene oxide. For single layer GO, the 2D band occurs as a single peak and the ratio will be seen to be equal to 2. An increase in the number of layers reduces the intensities of the 2D peaks. In the spectra of the analyzed materials, the ratio I_2D_/I_G_ for all samples is between 0.18 for GO-cembra pine and 0.27 for GO-limonene, these results indicating that GO materials are in the form of multilayers [74,75].

### 4.3. Thermogravimetric Analysis of Graphene Oxide Loaded with Active Compounds

All samples show a loss of mass at the beginning around 12%–13%, except for samples with gallic acid and limonene which have a mass loss of about 16%. This may be due to the fact that graphene oxide can easily absorb water and therefore when heated will lose it, together with other volatile compounds that are eliminated in this phase (RT-145 °C).

For graphene oxide (Figure 7a and Figure S1) the mass loss is 13.06%. Oxidation of the sample occurs suddenly (almost explosively), vigorously at ~155 °C, when the sample loses 60.05% by mass (in the range 145–165 °C) as indicated by the very sharp exothermic effect with the maximum at 155.3 °C. This process can be attributed to the loss of labile oxygen groups like epoxy or hydroxyl. Pyrolysis is specific to graphene oxide even if the atmosphere is inert because it is made with the oxygen contained in it. Then there are two slower oxidation steps, 165–460 °C, with a mass loss of 6.27% and 460–660 °C with a mass loss of 14.75%. These oxidations are accompanied by weak exothermic effects with a maximum at 189, 517.7, 604.4 and 631.6 °C respectively. The effect from 189 °C and the slow mass loss between 165–460 °C represent the removal of some slightly more stable groups like carbonyl or quinine [76,77]. After 500 °C, the burning of the carbon skeleton starts. For all samples, the analysis indicates the existence of a residual mass, in this case of 5.91% [78,79,80].

Thermal analysis for gallic acid sample (Figure 7b and Figure S2) indicates a more significant mass loss in the range of 30–145 °C (16.23%), similar to what has been observed so far, but more intense, mass loss being important even from 30 °C, the process is accompanied by a weak endotherm effect. This indicates a larger quantity of absorbed water in the sample due to the presence of gallic acid. Between 145 and 215 °C, an exothermic process takes place at a maximum of 186.6 °C, the associated mass loss being 20.33%. This process is less exothermic, and the corresponding mass loss is only one third from that of GO sample, indicating a chemical interaction between gallic acid and GO, not only a physical absorption. At least some of the labile oxygen groups are interacting with gallic acid molecules by condensation or hydrogen bonding and cannot be removed easily anymore. The sample then slowly loses mass up to 365 °C (6.92%) due to more stable oxygen groups found in GO. The simple gallic acid should melt at 256–260 °C when it is losing one molecule of water. However, there is no noticeable thermal effect in this region, confirming the fact that gallic acid is chemically bonded by GO and therefore its stability is enhanced [81]. In the range of 365 and 560 °C complete oxidation takes place, the mass loss being of 50.18%, the process is accompanied by a strong exothermic effect, divided into two, with a maximum at 419.5 and 464.5 °C, respectively. At this stage, there are at least two chained oxidation processes partially overlapped, most probably oxidation of the gallic acid residue and the oxidation of the carbon skeleton of GO [82]. 

Figure 7c (Figure S3) indicates that caffeic acid loaded into graphene oxide shows a slow weight loss (12.29%) between room temperature (RT) and 180 °C, like graphene oxide sample, the process is accompanied by a weak endothermic effect. Between 180 and 240 °C, an exothermic process (oxidation) takes place at a maximum of 221.3 °C, the associated weight loss being 22.4%. The sample then slowly losses mass up to 460 °C (11.27%), without any noticeable effect on the DSC curve. In the range of 460 and 640 °C complete oxidation takes place, the mass loss being 48.52%, the process is accompanied by a strong, asymmetric, exothermic effect, with the maximum at 554.1 °C. Overall the analysis is similar to the previous sample GOGA, the stabilization effect from the caffeic acid-GO bonds being slightly higher.

The thermal analysis for limonene sample (Figure 7d and Figure S4) indicates a mass loss of 16.01% in the range of 30 and 180 °C, similar to that observed in the GO samples, the process is accompanied by a weak endothermic effect. As limonene is an aliphatic hydrocarbon, it cannot generate strong bonds with GO, and therefore this sample behaves almost like a simple GO. Between 180 and 225 °C, a violent exothermic process took place with a maximum of 213.5 °C, the associated mass loss being of 65.03%. The sample is then relatively stable up to ~400 °C when the oxidation process of carbon skeleton starts. Up to 580 °C, 15.19% of the mass is lost, the process is accompanied by an exothermic split effect with maximums at 509.5 and 542.8 °C, respectively, indicating the existence of at least two partially overlapped oxidation processes. 

Thermal analysis for essential nutmeg oil sample (Figure 7e and Figure S5) shows a slow weight loss (12.53%) between RT and 175 °C, due to the elimination of water adsorbed in the sample, as well as other volatile molecules, similar to that seen with simple graphene oxide and the rest of the samples, the process is accompanied by a weak endothermic effect. Between 175 and 240 °C, there is an exothermic effect with a maximum at 220.1 °C, the associated mass loss being 23.25%. The main component of nutmeg oil is terpinen-4-ol which can condensate and generate hydrogen bonds with GO, therefore the thermal behavior of this sample being similar to those with gallic and caffeic acids. The sample slowly loses the mass up to 490 °C (12.61%). Between 490 and 640 °C, complete oxidation takes place, the mass loss being 45.97%, the process is accompanied by a strong, broad, exothermic effect, with the maximum at 590.7 °C. 

The sample loaded with cembra pine essential oil has a slow weight loss (12.35%) between RT and 180 °C, the process is accompanied by a weak endothermic effect, as shown in Figure 7f (Figure S6). Between 180 and 250 °C there is an exothermic process (oxidation) with a maximum of 231.5 °C, the associated mass loss being 23.54%. The main components of cembra pine essential oil are α-terpineol and some cyclic terpene alcohols. Therefore, the sample will behave similar to gallic acid–GO and caffeic acid–GO samples, as the bonds formed between essential oil and GO are by condensation and hydrogen bonds. The sample slowly loses mass up to 490 °C (12.06%) without any noticeable effect on the DSC curve. In the range of 490 and 640 °C complete oxidation takes place, with 45.84% mass loss, the process is accompanied by a strong exothermic effect, with the maximum at 588.6 °C.

As a conclusion, all samples present a first mass loss up to 145–180 °C due to the elimination of adsorbed water and some volatile molecules. A second mass loss occurs up to 165 °C for GO sample or up to 215–250 °C for the other samples and represents the loss of labile oxygen groups. The GO and limonene-GO sample undergo an energic oxidation process in this region, with a mass loss of at least 60%, indicating little chemical modification by limonene. Nevertheless, the limonene load on GO has the effect of increasing the thermal stability, the violent oxidation process taking place at 155 °C in the case of simple GO sample, but at 213 °C in the case of the limonene-GO sample. We can also observe that the limonene sample has no mass loss till 400 °C while GO sample presents a slow mass loss after 165 °C, up to 460 °C. Therefore, we can state that while limonene stabilizes the GO up to 213 °C, it will also generate the elimination of all GO oxygen groups in only one process. 

The gallic acid, caffeic acid, nutmeg and cembra pine essential oil samples contain molecules with -OH moiety and therefore can form hydrogen bonds and can condensate with oxygen groups from GO. The second mass loss process for these samples only represent around 20%–23%, and the exothermic effect is broader and less intense. It can be attributed to a slow oxidation process rather than an explosive one.

The last mass loss for all samples is the burning of the carbon skeleton of GO and oxidation of carbonaceous residue from the acids and oils loaded on GO. The processes take place over 400 °C usually and can be overlapped partially or totally giving a broad, intense, sometimes split exothermic effect. For the samples GO and limonene GO where large quantities of carbon were oxidized at a lower temperature, the thermal effect generated by burning of carbon skeleton is less intense. 

### 4.4. Release Behavior of Active Compounds

In comparison to gallic acid release behavior, the release rate of the other biologically active agents (caffeic acid, limonene, nutmeg and cembra pine essential oils) loaded onto graphene oxide is immaterial due to very low solubility and the content of the active substance released in the solution is below the limit of detection of used method [43,83].

The delivery of the gallic acid is quite fast on the first day (a burst delivery) followed by a strong decrease in the delivery for the next days. The cumulative release of gallic acid is about 80% after 10 days and practically is not changed in the next 20 days. These results have potential for GOGA platforms to be used as a drug delivery system having the ability to assure sustained release for over 10 days, as shown in Figure 8 [43]. 

### 4.5. Scanning Electron Microscopy (SEM)

The structure of the graphene oxide appeared in very thin agglomerates sheets, as can be seen at 1000X magnification in Figure 9. At higher magnifications, it can observe the waved and folded shape of the graphene oxide, the results being similar to those obtained and presented in other studies in the literature [41,84,85]. The agglomerates of graphene oxide visualized at 1000X exhibit a size up to a hundred micrometers and, at increasing magnifications the lamellar morphology of graphene oxide can be observed. Due to the high degree of functionalization, the characteristic sheets are not planar. 

As GO has a lamellar layer structure it is also possible to measure the width of the GO sample using SEM analysis. The individual GO sheets were found to have less than 20 µm being in good agreement with the literature [86].

The morphology of graphene oxide loaded with active compounds (Figure 10) does not show important changes, having the same general aspect as the graphene oxide sample because of the relatively low content of the loaded agents as well as due to the low gluing capacity of these agents. However, a slight increase in the degree of agglomeration of the particles due to the presence of the active compounds onto the graphene oxide support can be observed, giving them a slightly irregular and enlarged form.

### 4.6. Transmission Electron Microscopy (TEM)

TEM images showed GO flakes quite flat because of the suspension in a liquid. However, on the TEM grid were still found agglomeration of layers of different sizes, the thickness of the graphene oxide flakes being estimated to be ~4 nm. These findings are in accordance with the literature data [86,87]. The sheet with higher transparency indicates much thinner films of a few layers of GO due to a better exfoliation [88]. In order to explore the interlayer spacing of GO sheets, TEM analyses showed (Figure 11b) an interlayer spacing of investigated area about 2.12 Å (0.212 nm) indicating the presence of oxygen-containing functional groups (lower 2θ in GO than graphite) and are in agreement with the literature. Moreover, the transversal HRTEM image, Figure 11c, reveals a graphene oxide sheet of ~4.2 nm in thickness with a highly disordered surface arrangement of the atoms while the inner distribution of the atoms is much better because the surface oxidation is much higher [86,89]. Even if the arrangement of the atoms is not regular in cross-section view, the number of layers can be estimated to be between 10 and 20 layers.

## 5. Conclusions

The aim of the present work was to obtain graphene oxide-based drug delivery systems by loading caffeic acid, gallic acid, limonene, nutmeg and cembra pin essential oils starting from the corresponding solution. The SEM characterization showed the morphology of the graphene oxide support, which, compared to the loaded samples, a slight increase in the degree of agglomeration of the particles was observed due to the presence of the active compounds onto the graphene oxide samples. Raman spectra showed that no degradation occurred during loading. The ratio between I_2D_/I_G_ for all samples was between 0.18 for GO-cembra pine and 0.27 for GO-limonene; these results indicating that GO materials are in the form of multilayers. The individual GO sheets were found to have less than 20 µm. The thickness of GO flakes was estimated to be ~4 nm with an interlayer spacing of about 2.12 Å. 

According to the solubility of loaded biologically active agents, the release behavior of the selected biologically active agents was as follows: gallic acid release within 10 days can reach 80% but all the other biologically active agents are practically not released because their affinity for the graphene oxide support is higher than that to phosphate buffer solution. Further analyses are necessary to make biological assessments, both in vitro and in vivo. This results can be exploited accordingly, the systems which are not able to release the biologically active agents being suitable for certain applications (such as preventing infections, cancer spreading inside the grafts containing graphene oxide, etc.) while the systems releasing biologically active agents can be exploited in a classical way, as a platform for delivery of biologically active agents (especially in the treatment of cancer and severe infections). All these results encourage research towards the next level by carrying out in vitro and in vivo studies to demonstrate the ability of these platforms in the treatment of cancer and severe infections and in development of drug delivery systems and optical biosensing.

## Figures and Tables

**Table 1:** Biologically active agents used in the development of the graphene oxide-based platforms.

Active Substance	Biological Activity	Ref.
3,4,5-trihydroxybenzoic acid (gallic acid)	-antioxidant -anti-inflammatory -antibacterial -antimutagenic -antiviral	[35,42,43,44]
3- (3,4-dihydroxyphenyl) -2-propenoic acid (caffeic acid)	-antioxidant -antibacterial -cardiovascular -antimutagenic -anticancer -anti-leukemic -immunomodulatory	[45,46]
4-isopropenyl-1-methylcyclohexane (D-limonene)	-antioxidant -antimicrobial -anti-tumor -antidiabetic	[47,48,49,50]
Essential oils	-antimicrobial -anticancer	[51,52,53]

## Supplementary Materials

The following are available online at https://www.mdpi.com/1010-660X/55/6/230/s1, Figure S1: Thermogravimetric analysis of graphene oxide, Figure S2: Thermogravimetric analysis of graphene oxide loaded with gallic acid, Figure S3: Thermogravimetric analysis of graphene oxide loaded with caffeic acid, Figure S4: Thermogravimetric analysis of graphene oxide loaded with limonene, Figure S5: Thermogravimetric analysis of graphene oxide loaded with nutmeg essential oil, Figure S6: Thermogravimetric analysis of graphene oxide loaded with cembra pine essential oil.
