# Supplementary material for: Multifunctional Platforms Based on Graphene Oxide and Natural Products
Source: Medicina (Kaunas). 2019 May 30;55(6):230. doi: 10.3390/medicina55060230 (PMC6631192; doi:10.3390/medicina55060230)
Supplement: Supplementary file 1 [file medicina-55-00230-s001.pdf]

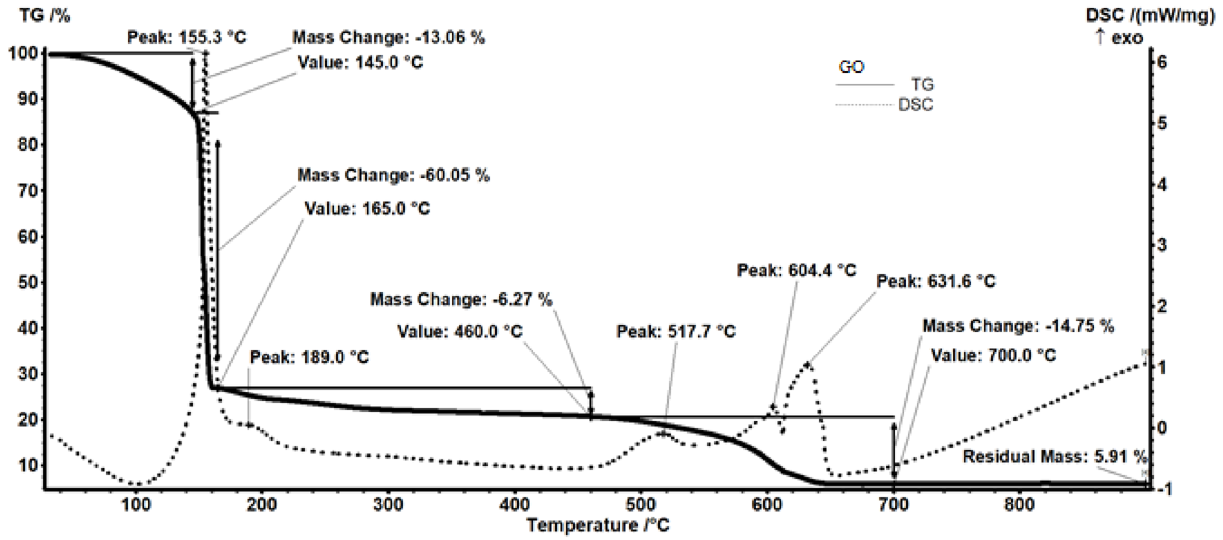

Figure S1. Thermogravimetric analysis of graphene oxide.

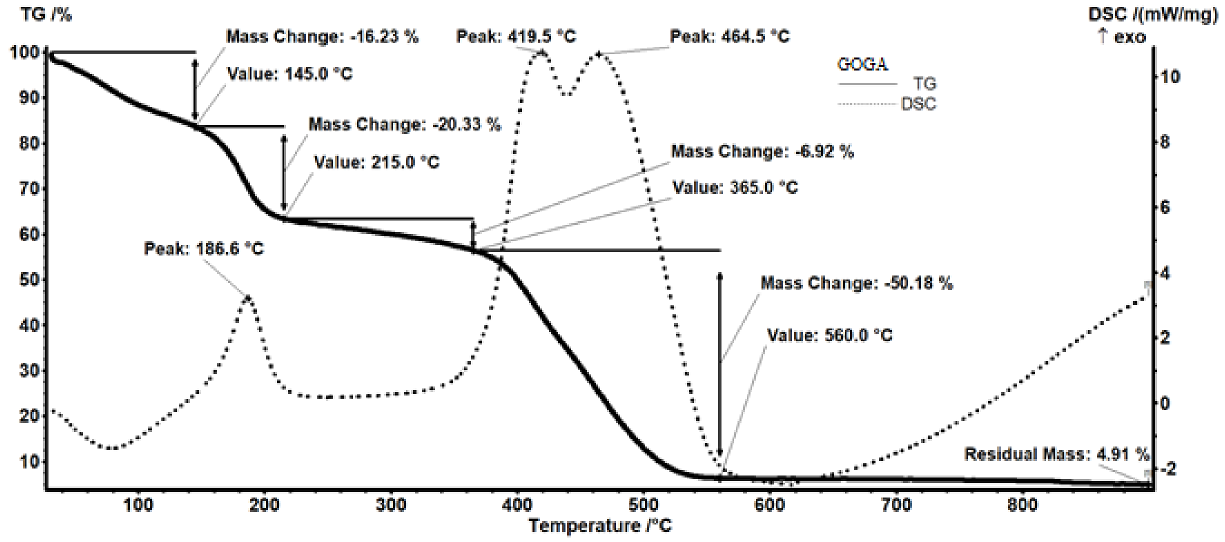

Figure S2. Thermogravimetric analysis of graphene oxide loaded with gallic acid.

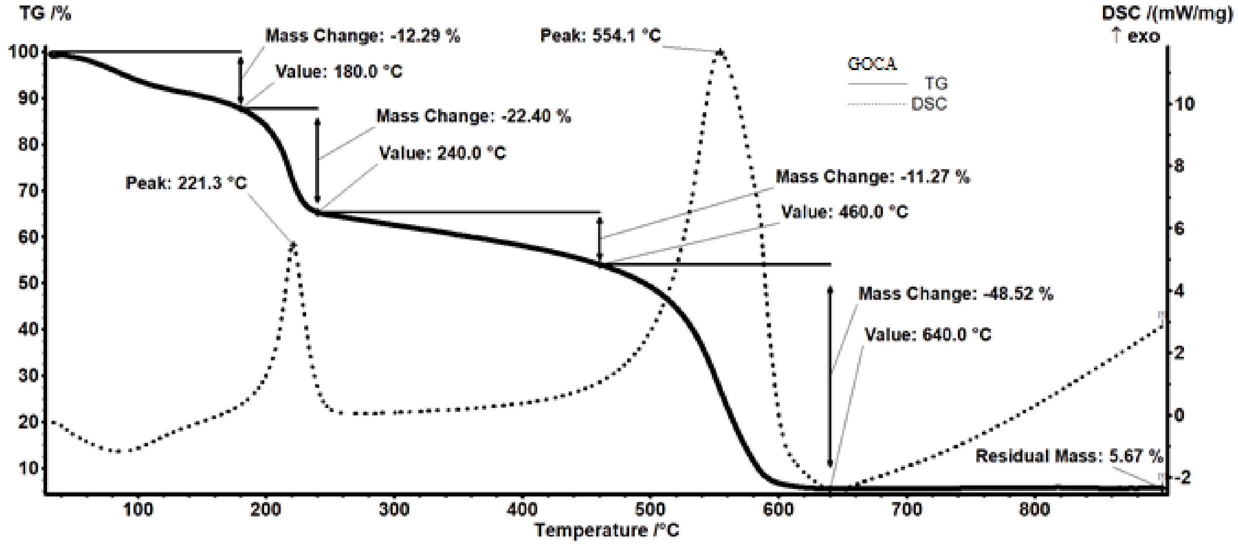

Figure S3. Thermogravimetric analysis of graphene oxide loaded with caffeic acid.

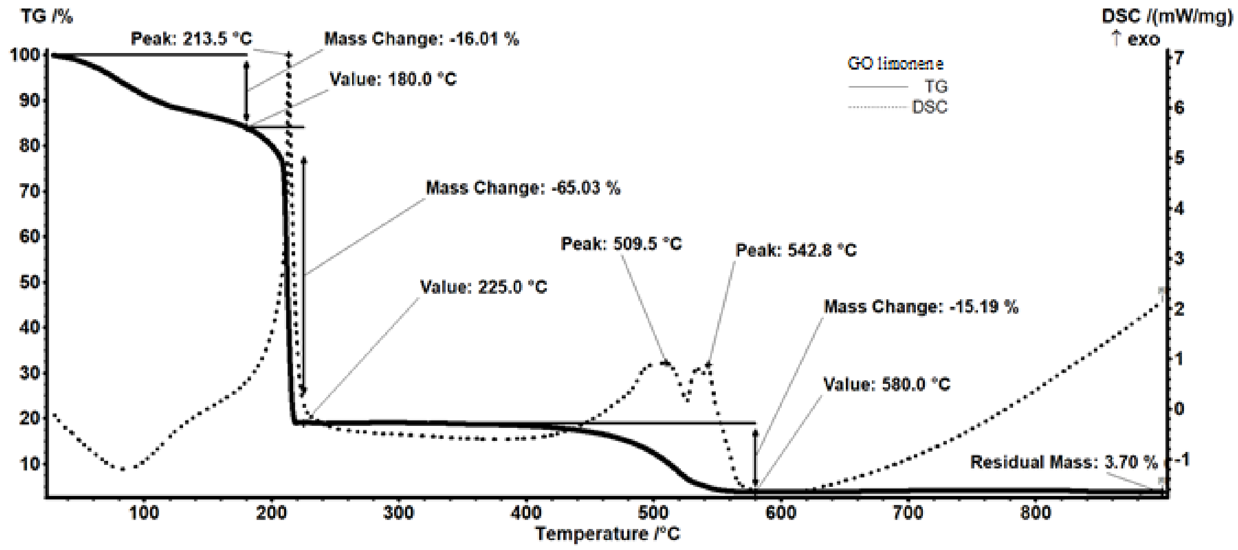

Figure S4. Thermogravimetric analysis of graphene oxide loaded with limonene.

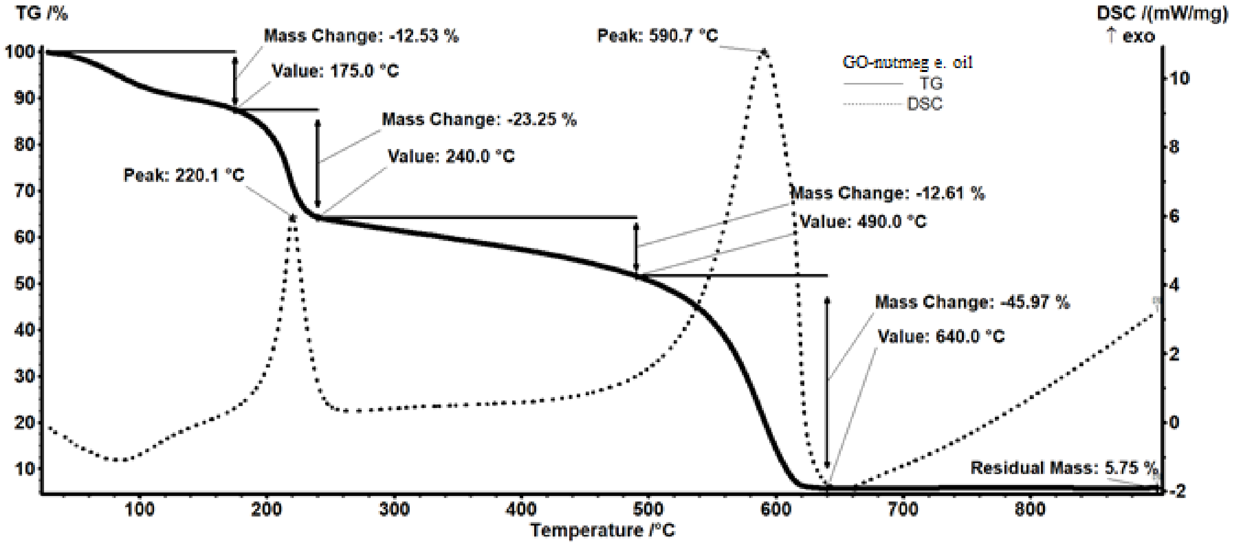

Figure S5. Thermogravimetric analysis of graphene oxide loaded with nutmeg essential oil.

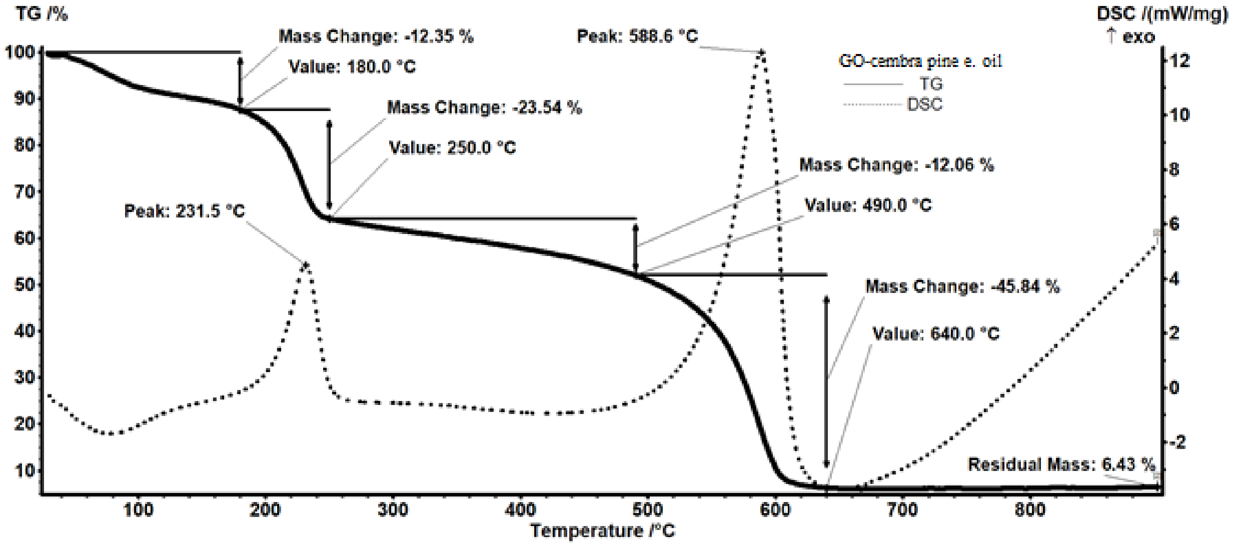

Figure S6. Thermogravimetric analysis of graphene oxide loaded with cembra pine essential oil.
